# Quality of Life and Volume Reduction in Women with Secondary Lymphoedema Related to Breast Cancer

**DOI:** 10.1155/2015/586827

**Published:** 2015-12-30

**Authors:** Marcus Lanza, Anke Bergmann, Maria Giseli da Costa Leite Ferreira, Suzana Sales de Aguiar, Ricardo de Almeida Dias, Karen de Souza Abrahão, Ester M. Paltrinieri, Ruy G. Martínez Allende, Mauro Figueiredo Carvalho de Andrade

**Affiliations:** ^1^Centro Universitário Augusto Motta, Rio de Janeiro, Brazil; ^2^Instituto Nacional de Câncer, Rio de Janeiro, Brazil; ^3^Centro Vodder Argentina, Buenos Aires, Argentina; ^4^Faculdade de Medicina da Universidade de São Paulo, São Paulo, Brazil

## Abstract

*Purpose*. To assess the quality of life (QOL) as a predictor of volume reduction in women undergoing complex physical therapy (CPT) for lymphoedema following breast cancer.* Methods*. Clinical trial in 57 women undergoing CPT.* Results*. At baseline, in measuring quality of life for the EORTC QLQ-C30 questionnaire subscale of functionality, the worst scores for emotional function (55 points) and better social function (89 points) were observed. The symptom scales showed the worst pain averaged (66 points). The overall quality of life showed a low score (40 points). In the BR 23 module, low scores were observed in the field of future perspective (47 points). After treatment of lymphoedema, absolute reduction of excess volume between the upper limbs of 282 mL was observed, representing a reduction of 15%. No association was observed between the domains of quality of life and response to treatment of lymphoedema.* Conclusion*. This study included 57 women with advanced and chronic lymphoedema in early treatment with CPT and low scores for quality of life. The lymphoedema therapeutic response was not influenced by the QOL at the beginning of treatment.

## 1. Introduction

Breast cancer has become a major focus of worldwide attention due to the increase in incidence over the past decades. In Brazil, it is an important public health problem, being the most common neoplasm among women and the leading cause of death from cancer in this population group. In 2014, 57,120 new cases were estimated representing a rate of 56.09 cases per 100,000 women [1].

In Brazil, the issue of breast cancer is compounded by the frequency of diagnoses at more advanced stages of the disease. On average, 60% of cases are diagnosed at stages III and IV, leading to more aggressive and mutilating surgeries, increasing the incidence of complications arising from oncological treatment [2, 3].

Lymphoedema is a major complication of treatment for breast cancer, reaching, in our population, about 30% of women after five years of postoperative follow-up [4].

Several approaches to the treatment of lymphoedema have been reported in the literature [5, 6]. Among conservative treatments, complex physical therapy (CPT) has emerged as the best approach to control the volume of lymphoedema of the upper limb [7–14]. The first phase of treatment aims for the maximum reduction in limb volume and is part of this treatment: skin care, manual lymph drainage (MLD), exercises, and compression bandaging. Immediately after this phase, the maintenance phase (second phase) starts, consisting of adaptation of compression garments, exercise, and self-massage, with the goal of preserving and optimising the results obtained in the initial phase [10–13].

The therapeutic response to any treatment provided depends not only on the physiological actions of the treatment but also on the cultural, social, psychological, and physical conditions of each patient. One of these components, which may influence the therapeutic response, is the quality of life. According to the World Health Organization (WHO) quality of life is defined as “individuals” perception of their position in life in the context of culture, value systems in which they live in relation to their goals, expectations, standards, and concerns [15]. For patients with breast cancer, quality of life assessment will approach the way they experience changes caused by the disease as well as the positive or negative influence that the treatment will have on their lives [16–23].

In this context, this study aims to evaluate QOL as a predictor of treatment response in women undergoing CPT in the treatment of upper limb lymphoedema after axillary lymphadenectomy.

## 2. Materials and Methods

A randomised clinical trial was conducted in women with secondary treatment of breast cancer lymphoedema. The study methodology has been previously detailed [9].

In order to perform the study, 57 women referred for unilateral axillary dissection, with a difference between the upper limbs greater than three centimeters in circumference at one point at least, who were not undergoing chemotherapy or adjuvant radiotherapy and showed no heart disease or systemic decompensated hypertension, were included. Women were excluded if they had surgery less than six months previously, were diagnosed with preoperative lymphoedema, showed signs of inflammation in the swollen limb, had a history of allergic reaction to the material used for compression bandaging, had active locoregional or distant disease, or had been undergoing treatment for lymphoedema with compression bandaging for the last three months.

For the treatment of lymphoedema, all patients underwent complex physical therapy (skin care, compression bandaging, exercises, and home orientations), with or without manual lymphatic drainage.

The main outcome (therapeutic response) was considered to be the percentage reduction in excess limb volume between the beginning and the end of treatment (end of compressive bandaging, occurring in about 4 weeks), calculated by (*IV* − *FV*/*IV*)*∗*100, with *IV* being the initial volume and *FV* the final volume. The estimated volume of the limb (*V*) was obtained from the circumference. The measures for each point which were used were *V* = *h* *∗* (*C* ^2^ + *C∗c* + *c* ^2^)/(*π∗*12), in which *V* is the volume of the limb segment, *C* and *c* are the circumferences at each end, and *h* is the distance between the circumferences (*C*).

Descriptive variables were collected, such as age, body mass index (BMI), time to onset postoperative lymphoedema, excess volume in the segment, duration of chronic lymphoedema, and the number of infections in the limb with lymphoedema.

As the main predictor variable, the mean score of quality of life was considered, measured at study entry. Quality of life was assessed by the EORTC QLQ-C30 and BR23, both validated by the Brazilian population [16]. QLQ-C30 is a multidimensional questionnaire designed to assess the psychological and social functioning of patients with cancer. It is made up of 30 questions that assess five functional scales (physical, functional, cognitive, emotional, and social role), three symptom scales (fatigue, pain, nausea, and vomiting), and quality of life scales and overall health. QLQ-BR23 intends to specifically evaluate the effects of treatment in patients with breast cancer. It consists of 23 questions divided into two scales: physical functioning and symptoms. The functional scale is divided into ranges of body image, sexual function, sexual satisfaction, and future perspective. The second level consists of the subscales: adverse effects of systemic therapy, breast symptoms, and arm symptoms. QLQ-C30 and BR23 have a scale ranging from 0 to 100, in which 0 is the worst health status and 100 the best of health, except for the symptom scales in which higher scores represent more symptoms and worse quality of life. For statistical analysis, the symptom scale was reversed so that higher scores represent better quality of life [15].

The descriptive analysis of the study population was performed by using measures of central tendency and dispersion. To evaluate the changes in excess volume before and after treatment, we performed the Wilcoxon signed-rank test, considered statistically significant at *p* < 0.05. Linear regression was performed to evaluate the association between the scores of the domains of quality of life before treatment and the therapeutic response. The statistical package SPSS 20.0 was used for all analyses.

The project was approved by the Ethics Committee in Research of the National Cancer Institute (INCA) under registration number 011/07 and was in accordance with the Helsinki Declaration of 1975, as revised in 2008. All participants signed the informed consent form.

## 3. Results

The study included 57 women. At inclusion in the study, the average age of the study population was 63 years (SD 10.02), mostly overweight and obese. The average time after surgery until the development of lymphoedema was 37 months. At the beginning of treatment, lymphoedema was present for a median of 61 months (5 years). At baseline, the mean excess volume (EV) between the upper limbs was 776.16 mL, corresponding to a percentage excess volume (PEV) of 44.2% (Table 1).

After treatment, the absolute decrease in statistically significant excess volume between the upper limbs of 282 mL was observed, representing a reduction of 15% (*p* = 0.001) (Table 2).

In measuring quality of life for the functional scale of the EORTC QLQ-C30 questionnaire, the worst scores for emotional function (55 points) and better social function (89 points) were observed. In the symptom scales, pain presented the worst average (66 points), followed by fatigue (68 points). The overall quality of life showed a low score (40 points). When evaluating the quality of life by BR 23 module, a low score was observed in the field of future perspective (47 points) and arm symptoms (53 points). There was no association between the domains of quality of life and therapeutic response to treatment of lymphedema (Table 3).

## 4. Discussion and Conclusion

Our results showed that the quality of life at the beginning of treatment was not a predictor of therapeutic response in women undergoing CPT in the treatment of upper limb lymphoedema secondary to breast cancer. Different aspects need to be discussed so as to better understand these results, including the sociodemographic and clinical characteristics of women included in this study.

The women studied were from a single public institution, with reference to the treatment of breast cancer (Brazilian National Cancer Institute). Such women are given physical therapy at all stages of cancer treatment with the aim of preventing and minimising complications resulting from breast cancer treatment and its progression [24]. However, even with access to physical therapy, average excess volume of 44% between the upper limbs at the beginning of treatment was observed. Lymphoedema developed on average 37 months after surgery and was present on average 60 months previously. In another clinical trial with similar methodology conducted with 102 Canadian patients, in most cases (73%) the average excess volume of the affected limb was between 10% to 30% and lymphoedema was present for less than one year in 43% of patients [11]. In a study conducted with crossover design with 32 patients in the early intervention, 34.4% of cases were classified as severe lymphoedema (>40% excess volume) and the average duration of lymphoedema was 73 months [25]. Therefore, our population has more advanced and greater chronicity compared to previous lymphoedema studies.

The use of quality of life assessments in breast cancer patients has an important role as a risk for complications, treatment outcome, and prognosis [19, 22, 23, 26]. With regard to quality of life before physiotherapy, when assessed by the EORTC QLQ C-30 instrument, the average for overall quality of life was 40.2. In the functional domain, worse scales for quality of life were in emotional function (mean 54.82) and cognitive function (mean 63.43). On the scale of symptoms, worse quality of life scores were reported for pain (mean 65.7) and fatigue (mean 68.4). When assessing the quality of life by BR 23 module, a low score was observed in the domain of future perspective (47 points) and arm symptoms (53 points).

In a study performed by Gurdal et al. [8], using the EORTC QLQ C-30 instrument and also evaluating before treatment for lymphoedema, the population showed a better mean score of overall quality of life (58.2 and 64.8, depending on the group treatment). In the domain of functionality, the physical and social functions were the most affected and the symptoms of pain and fatigue had the worst score. The difference in the functionality of our patients may reflect cultural issues, especially those related to body image. Physical symptoms associated with lymphoedema include decreased strength and range of motion of the upper limb, fatigue, and pain. These symptoms, associated with the appearance of lymphoedema, can lead to negative self-image, especially in relation to body image, which also affects social and sexual relationships. Emotional well-being is also altered, and these women experience greater stress, anxiety, sadness, anger, frustration, and guilt in relation to their situation [27].

Regarding the response to lymphoedema treatment, several clinical trials and observational studies which have performed CPT report a statistically significant decrease in limb volume before and after treatment [11–14, 28–30]. In our population, the average reduction in excess volume was 282 mL, with 15% reduction (*p* < 0.001). The best result was observed in a randomised clinical trial of 77 women treated with compression bandaging, exercises, and MLD group. A reduction rate of 36% and 56% was reported (according to treatment group) [12]. This difference in treatment response can be explained by the protocols established in each service. In our population, as it is within a public hospital with great demand for lymphoedema treatment, patients were treated twice a week, using compression bandaging throughout the period, which may explain the worse therapeutic response.

In this study, we found no association between quality of life at the beginning of treatment and the therapeutic response of the CPT in any studied domain. Neither did we find in the literature other studies that have evaluated these variables.

This study has some limitations, among which we can highlight the sample size which may have influenced the lack of association between the therapeutic response and the quality of life symptoms subscale in the arm symptoms subscale (*p* = 0.099). In other scales evaluated, it is unlikely that an increase in the sample size would change the result. As strengths, we highlight the homogeneity of women and little possibility of selection and classification bias, since the data were collected from patients from a single institution in reference to the treatment of breast cancer, which gives adequate internal validity of the study. However, generalization of the results (external validity) should be viewed with caution. Studies in other populations with different treatment and sociodemographic conditions could be conducted to better understand the influence of quality of life as a predictor of therapeutic response of lymphoedema.

In conclusion, this study included 57 women with advanced and chronic lymphoedema in early treatment with CPT. Domain scores of quality of life observed at inclusion of the study had worse scores for emotional function and better scores for social function. In symptom scales, pain and fatigue presented the worst average (68 points). Overall quality of life scores were low. When evaluating the quality of life by BR 23 module, a low score was observed in the field of future perspective and arm symptoms. After treatment, absolute reduction of excess volume between the upper extremities of 282 mL was observed, representing a reduction of 15%. No association was observed between the domains of quality of life and therapeutic response to treatment of lymphoedema.

## Figures and Tables

**Table 1:** Characteristics of patients before the intervention.

Variables	Mean (SD)	Median (min.–max.)
Age (in years)	62.87 (10.02)	63.95 (39–88)
Time (months) postoperatively for lymphedema	37.47 (55.60)	21.70 (1.10–309.47)
Excess volume (mL)	776.16 (490.19)	652.12 (158–2.271)
Percentage of excess volume	44.20 (26.83)	38.17 (7.87–139.91)
Time (months) with chronic lymphedema	60.90 (62.98)	50.77 (0–318)
Body mass index	29.75 (5.57)	29.40 (21–48)

SD: standard deviation.

**Table 2:** Response therapeutic for lymphedema treatment.

Volume of the UL	Before treatment	After treatment	Difference (before, after)			Wilcoxon signed-rank test			
			Mean	95% CI		Negative^a^ *N*	Positive^b^ *N*	Unchanged^c^ *N*	*p* ^*∗*^
	Mean	Mean		Lower	Higher				
Excess volume between the upper limbs	776.16	494.51	281.65 mL	218.57	344.73	56	1	0	<0.001
Percentage of excess volume between the upper limbs	44.20%	29.18%	^*∗∗*^15.02%	11.52	18.53	53	4	0	<0.001

^*∗*^The mean difference between the beginning and end of treatment was analyzed using Wilcoxon test; ^*∗∗*^Kolmogrov-Smirnov test *p* > 0,05.

^a^Volume tracking < initial volume. ^b^Volume tracking > initial volume. ^c^Volume tracking = initial volume.

CI: confidence interval; *N*: number of patients.

**Table 3:** Linear regression model between the score of quality of life at baseline and percentage reduction of excess volume between the upper limb after treatment.

Quality of life	Mean (SD)	Simple linear regression	
		Beta (CI 95%)	*p* value
*EORTC QLQ C30*			
Functional scale			
Physical function	70.39 (21.39)	−0.05 (−0.22–0.12)	0.591
Performing roles	71.16 (33.50)	0.03 (−0.07–0.14)	0.520
Cognitive function	63.43 (28.95)	0.01 (−0.11–0.14)	0.826
Emotional function	54.82 (31.31)	−0.05 (−0.17–0.06)	0.388
Social function	89.42 (19.54)	−0.01 (−0.20–0.18)	0.917
Symptoms scale			
Dyspnea	82.70 (33.31)	0.05 (−0.05–0.16)	0.318
Pain	65.72 (35.90)	0.00 (−0.10–0.10)	0.992
Fatigue	68.37 (30.83)	−0.03 (−0.15–0.08)	0.563
Insomnia	76.29 (36.29)	−0.04 (−0.14–0.05)	0.372
Loss of appetite	92.30 (19.38)	0.01 (−0.17–0.20)	0.878
Nausea and vomiting	92.63 (14.54)	0.09 (−0.16–0.34)	0.486
Constipation	83.33 (31.30)	−0.04 (−0.15–0.08)	0.505
Diarrhea	94.87 (19.10)	−0.02 (−0.21–0.17)	0.863
Financial hardship	76.94 (38.20)	0.07 (−0.02–0.16)	0.139
*Overall quality of life*	40.20 (29.18)	0.02 (−0.10–0.15)	0.728
*BR23*			
Functional scale			
Body image	76.37 (29.02)	0.01 (−0.11–0.14)	0.826
Future perspective	47.28 (40.90)	−0.03 (−0.12–0.05)	0.448
Sexual function	76.06 (25.00)	0.00 (−0.14–0.15)	0.951
Symptoms scale			
Systemic therapy	75.24 (20.23)	0.08 (−0.10–0.25)	0.385
Arm symptoms	53.34 (27.16)	−0.11 (−0.24–0.02)	0.099
Breast symptoms	83.49 (21.36)	−0.00 (−0.17–0.17)	0.994
